# Development and validation of a novel mitophagy-related gene prognostic signature for glioblastoma multiforme

**DOI:** 10.1186/s12885-022-09707-w

**Published:** 2022-06-13

**Authors:** Jinghua Wang, Xinqi Qiu, Jiayu Huang, Zewei Zhuo, Hao Chen, Ruijie Zeng, Huihuan Wu, Kehang Guo, Qi Yang, Huiling Ye, Wei Huang, Yujun Luo

**Affiliations:** 1 Department of Hematology, Guangdong Provincial People’s Hospital, Guangdong Academy of Medical Sciences, Guangzhou, Guangdong China; 2 Zhuguang Community Healthcare Center, Guangzhou, 510080 China; 3 Department of Otorhinolaryngology-Head and Neck Surgery, Huizhou Municipal Central People’s Hospital, Huizhou, 516001 People’s Republic of China; 4 School of Bioscience and Bioengineering, South China University of Technology, Guangzhou, 510006 China; 5 Department of Gastroenterology, Guangdong Provincial People’s Hospital, Guangdong Academy of Medical Sciences, Guangzhou, Guangdong 510080 People’s Republic of China; 6 Department of General Medicine, The First Affiliated Hospital of Guangzhou Medical University, Guangzhou, 510120 China; 7 Department of Pathology and Laboratory Medicine, University of Cincinnati College of Medicine, Cincinnati, OH 45267 USA

**Keywords:** Mitophagy, Risk model, Glioblastoma multiforme, Prognosis, Risk signature

## Abstract

**Background:**

Glioblastoma multiforme (GBM) is one of the most malignant tumors in brain with high morbidity and mortality. Mitophagy plays a significant role in carcinogenesis, metastasis, and invasion. In our study, we aim to construct a mitophagy-related risk model to predict prognosis in GBM.

**Methods:**

RNA-seq data combined with clinical information were downloaded from TCGA. The 4-gene risk model and nomograph was then constructed and validated in external cohort. Evaluation of immune infiltration, functional enrichment and tumor microenvironment (TME) were then performed.

**Result:**

A mitophagy-related risk model was established and patients in TCGA and CGGA were classified into low-risk and high-risk groups. In both cohorts, patients in low-risk group had improved survival, while high-risk group had poor prognosis. Also, the risk model was identified as an independent factor for predicting overall survival via Cox regression. Furthermore, a prognostic nomogram including mitophagy signatures was established with excellent predictive performance. In addition, the risk model was closely associated with regulation of immune infiltration as well as TME.

**Conclusion:**

In conclusion, our study constructed a mitophagy-related risk model, which can be utilized for the clinical prognostic prediction in GBM.

**Supplementary Information:**

The online version contains supplementary material available at 10.1186/s12885-022-09707-w.

## Introduction

Gliomas are the most common brain tumors in central nervous systems and represent 75% malignant brain cancer in adults. Glioblastoma (GBM), also known as glioblastoma multiforme, is the most lethal type in gliomas (World Health Organization [WHO] grade IV), with a 5-year relative survival of 3 ~ 7% [1, 2]. Despite of the progress in multimodality therapies including surgical resection, radiotherapy, systemic therapies (chemotherapy, targeted therapy) and supportive care, the median survival time of GBM is less than 2 years [3]. These conditions suggest that the conventional stage system for predicting prognosis such as WHO grade is insufficient to cover the clinical diversity of GBM. Thus, a novel prognostic model for GBM is needed to be established.

The role of autophagy in malignant metastasis and response to treatment has been elucidated in many types of tumors, which is characterized by degrading dysfunctional and cellular materials or damaged organelles through lysosomal system to maintain cellular homeostasis [4, 5]. Mitophagy, as a specific type of autophagy, is a fundamental process of removing damaged or excessive mitochondria via autophagolysosomes. This process is often triggered by oxidative stress or increasing need of bioenergy, which is necessary for cancer formation and invasion [6]. Some studies have discovered the role of mitophagy in gliomas [7, 8]. Huang et al. found that inhibition of mitophagy partially reverted cannabidiol-induced glioma cell death, suggesting the positive role of mitophagy on anti-tumor [9]. Two studies demonstrated that induction of mitophagy by FOXO3a protect the gliomas from temozolomide-induced cytotoxicity, indicating double-sword effect of mitophagy on glioma [10, 11]. Thus, whether mitophagy is correlated to the prognosis of GBM, and the possible involvement of mitophagy-related genes remain to be explored.

Given the existing findings, we constructed a mitophagy-related prognostic model based on mRNA expression and clinical data of GBM patients from TCGA, and we also explored the correlations between mitophagy and the tumor immune microenvironment.

## Materials and methods

### Datasets

RNA sequencing (RNA-seq) data of 169 GBM patients with 5 normal controls were extracted from TCGA database on 1 August 2021 (https://portal.gdc.cancer.gov/repository). The validation data were extracted from CGGA database mRNAseq_693 on 1 August 2021 with a total of 134 GBM patients [12, 13] (http://www.cgga.org.cn/).

### Identification of mitophagy-related differential expressed genes (DEGs)

Fifty-one mitophagy-related genes were extracted from Gene Set Enrichment Analysis (http://www.gsea-msigdb.org/gsea/index.jsp) combined with GeneCards (https://www.genecards.org/). Then mRNA expression data were compared utilizing “limma” package between 169 GBM patients and 5 controls [14]. DEGs were screened out with the following cut off: *P* value < 0.05. PPI network of DEGs was constructed using Search Tool for the Retrieval of Interacting Genes (STRING), version 11.0 (https://string-db.org/).

### Establishment and validation of risk model for prognosis

To further screen out DEGs with high predicting value, we performed Cox regression analysis to evaluate the relation between DEGs and survival status in TCGA cohort. 5 mitophagy-related genes were firstly identified for further analysis with *P* < 0.1. LASSO regression analysis was performed via R package “glmnet” to identify prognosis-related DEGs and developed risk model [15]. Finally, 4 genes were selected as the optimal gene to construct risk score. The calculation of risk score was as follows: Risk Score = ∑ ${\sum}_i^4 Xi\times Yi$ (X: coefficients, Y: gene expression level). According to median value of the risk score, patients in TCGA were well classified into low-risk and high-risk groups and the survival status of 2 groups were further compared using Kaplan–Meier analysis. PCA of 4-genes risk model was constructed by R package “Rtsne”. The time-dependent ROC curve was applied to evaluate the predicting efficacy of risk score utilizing R “survivalROC” package [16]. The images of immunohistochemistry (IHC) staining of prognosis-related genes were extracted from The Human Protein Atlas database [17]. To analyze independent prognosis value, univariate and multivariable Cox regression models were employed in TCGA cohort and CGGA cohort. To validate the risk model, CGGA cohort (mRNAseq_693) was applied. 4-genes risk signature was then calculated by the same method in TCGA cohort and patients in CGGA were divided into low-risk and high-risk groups and the difference was further analyzed according to method mentioned above. The nomograph was constructed using R package “rms”, “foreign”, “survival” and evaluated by ROC curve. The Calibration plots were applied to evaluate the discriminative ability of the nomogram.

### Functional enrichment based on GO and KEGG

Gene Ontology analysis was performed to enrich the biological processes, molecular functions and cellular components based on DEGs between low-risk and high-risk groups. And the Kyoto Encyclopedia of Genes and Genomes pathway analysis were conducted to determine the pathway corelated to the Mitophagy-related risk signature. GO and KEGG analysis both used R package “clusterProfiler” [18].

### Estimation of immune infiltration and immune related pathway

The scores of infiltrating immune cells and immune-related pathways were evaluated using R package “gvsa” in TCGA cohort and CGGA cohort [19]. Furthermore, the relationships between immune cells and mitophagy-related genes were analyzed through TIMER database [20] (https://cistrome.shinyapps.io/timer/).

### Tumor microenvironment analysis

The 22 types of immune cells composition in subgroups were analyzed using CIBERSORT algorithm (https://cibersort.stanford.edu/). The correlations between 22 immune cells composition were evaluated via “corrplot” R package**.**

### Statistical analysis

R software (version 4.1.0), SPSS (version 23.0) and R studio (version 1.1.463) were used to perform statistical analysis. Single-factor analysis was utilized to compare the different expression of genes. Person chi-square test was used to evaluate the categorical variables. Kaplan-Meier method combined with log-rank test was used to analyze the overall survival (OS) of GBM patients. Univariate and multivariate Cox regression were used to assess the independent prognostic value of the risk model. The analysis of immune cell infiltration and immune pathways were conducted via Wilcoxon test.

## Results

### Identifying prognostic-related DEGs that were associated with mitophagy

**Fig. 1:**
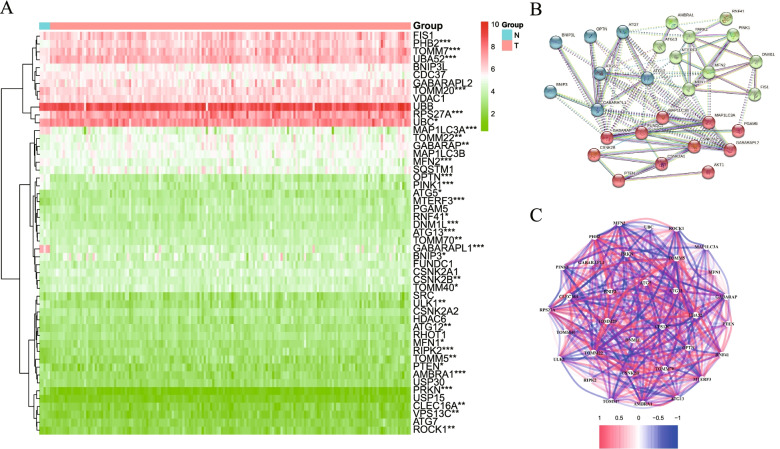
Expression pattern of 51 mitophagy-related genes between GBM and normal patients in TCGA cohort. **A** Heatmap indicating different expression genes (DEGs) between tumor (red) and normal sample (blue). ^*^ *P* < 0.05, ^**^P < 0.01, ^***^ *P* < 0.001. **B** PPI network for the interaction between mitophagy-related genes. (Interaction score = 0.9). **C** The correlation network of the 33 DEGs. (Red line: positive relation, blue line: negative relation)

A total of 5 normal and 169 GBM samples with mRNA expression profile and clinical data were extracted from TCGA. The 51 mitophagy-related genetic expressions were compared between normal group and tumor group via “limma” package. Twenty-seven mitophagy-related DEGs were identified with *P* < 0.05, among which 15 genes (*GABARAPL1, PRKN, OPTN, PINK1, MAP1LC3A, ULK1, DNM1L, AMBRA1, TOMM20, ATG13, MFN2, PTEN, TOMM70, RNF41, VPS13C*) were downregulated while 12 genes (*TOMM40, GABARAP, CSNK2B, TOMM22, TOMM5, ROCK1, TOMM7, RIPK2, MTERF3, UBA52, PHB2, RPS27A*) were upregulated. The heatmap of 51 DEGs was illustrated in Fig. 1A. To further explore the interaction between DEGs, we performed protein–protein interaction (PPI) on mitophagy-related gene (Fig. 1B). We identified 28 hub genes with the minimum required interaction score of 0.900 (highest confidence), among which 10 genes (*ATG 13, CSNK2B, DNM1L, GABARAP, MAP1LC3A, MFN2, MTERF3, OPTN, PINK1, PTEN*) were DEGs (Fig. 1B). The correlation network of 33 mitophagy-related genes expressions with significant difference is further shown in Fig. 1C (caption: red = positive correlations; blue = negative correlations).

### Consensus clustering analysis of GBM based on different expression pattern

**Fig. 2:**
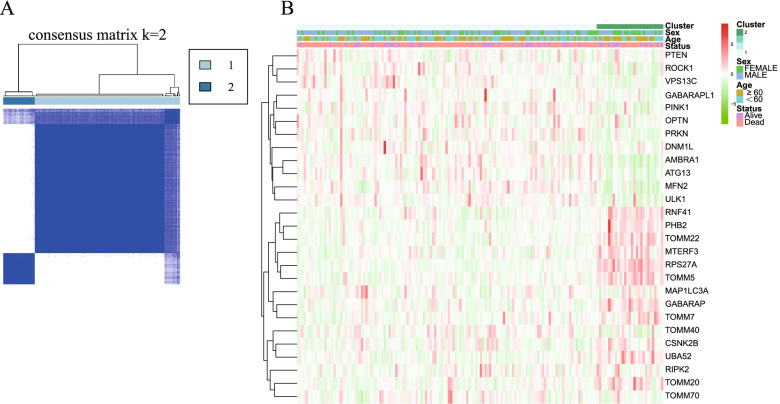
Classification of tumor samples based on mitophagy-related DEGs. **A** 159 GBM patients were separated into two clusters based on the consensus clustering matrix (*k* = 2). **B** Heatmap of two clusters with clinical information

To further classify GBM subtypes based on their distinct expression pattern of mitophagy-related DEGs, we constructed consensus clustering analysis in GBM patient in the TCGA cohort. By using ConsensusClusterPlus package based on 27 mitophagy-related DEGs, we identified 2 different regulation patterns (*k* = 2) including 139 cases in cluster 1 and 30 in cluster 2 with the highest intragroup correlations (Fig. 2A). Then we integrated the cluster with the mRNA expression level and clinical informations including age (≤60 or > 60 years), sex (male or female) and survival status (dead or alive), which are illustrated in heatmap (Fig. 2B).

### Construction of risk signature based on the TCGA cohort

**Fig. 3:**
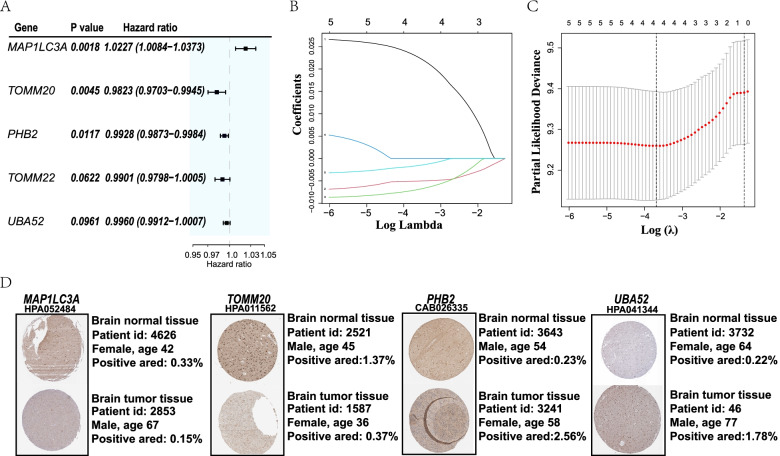
Construction of risk model in TCGA cohort based on mitophagy-related genes. **A** Univariate cox regression analysis of OS for 5 mitophagy-relation genes with *P* < 0.1. **B** least absolute shrinkage and selection operator **(**LASSO) regression of 5 OS-related genes. Black: *MAP1LC3A*, Blue: *TOMM 22*, Baby blue: *PHB2,* Red: *TOMM 20*, Green: *UBA52*. **C** Cross-validation in LASSO regression. **D** Immunohistochemistry staining of normal and tumor sample. Positive area was measured by image J

After deleting duplicate and missing value, a total of 159 GBM samples were match with clinical information. To screen out candidates for constructing risk signature, we performed univariate Cox regression analysis on DEGs and identified 5 genes (*MAP1LC3A*, *TOMM20*, *TOMM22*, *PHB2*, *UBA52*) with the criteria of *P* < 0.1 (Fig. 3A). Among them, 1 gene (*MAP1LC3A*) was related to increased risk with HRs > 1, while the other 4 genes (*TOMM20*, *TOMM22*, *PHB2*, *UBA52*) were associated with protective effect with HRs < 1 (Fig. 3A). Next, least absolute shrinkage and selection operator (LASSO) Cox regression analysis was used to identified 4 survival-related genes (*MAP1LC3A*, *TOMM20*, *PHB2*, *UBA52*) to construct risk model for prognosis according to the optimum λ value (Fig. 3B, C). The risk score formula was as follows: risk score = (0.0272 * *MAP1LC3A* exp.) + (− 0.00546 * *PHB2* exp.) + (− 0.008623 * *TOMM20* exp.) + (− 0.002654 * *UBA52* exp.). The downregulation of *MAP1L3A*, *TOMM20* and upregulation of *PHB2*, *UBA52* in GBM tissue were further validated by The Human Protein Atlas database (Fig. 3D).

**Fig. 4:**
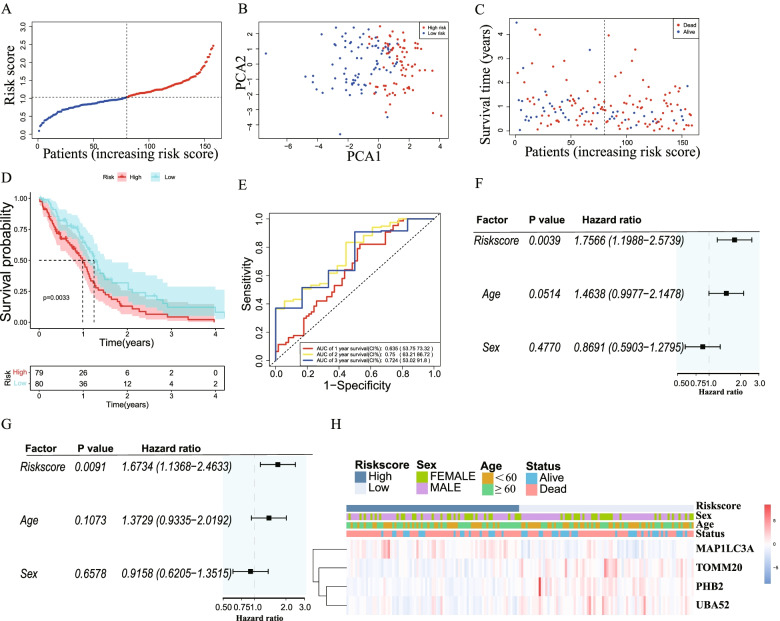
Predicting value of risk model in TCGA cohort. **A** Distribution of patients according to risk model. **B** Principal component analysis (PCA) plot of GBM patient based on risk model. **C** The survival status and time of patient with GBM. (Low-risk: on the left side of dotted line. High-risk on the right side of dotted line). **D** Kaplan-meier (KM) survival analysis of patients in low-risk and high-risk groups. **E** ROC curve for predicting efficiency of the risk model. **F** Univariate Cox regression analysis of risk score in TCGA cohort. **G** Multivariate Cox regression analysis of risk score in TCGA cohort. **H** Heatmap of patients in different risk group with clinical information. (Red: positive expression. Blue: negative expression)

The 159 samples were classified into high-risk group (79) and low-risk group (80) (Fig. 4A). Principal component analysis (PCA) showed that GBM patients were well divided into 2 clusters (Fig. 4B). As shown in Fig. 4C, patients in high-risk group exhibited more death (red dot) and lower survival time compared to low-risk group. Furthermore, the difference of OS between high-risk and low-risk groups was prominent (*P* = 0.0033) (Fig. 4D). Time-dependent ROC curves were utilized to evaluate the predictive performance of the model and the area under the curve (AUC) was 0.635 for 1-year, 0.75 for 2-year, and 0.724 for 3-year survival (Fig. 4E).

To evaluate whether risk score was an independent risk factor, we utilized univariate and multivariable Cox regression analyses in TCGA cohort. In univariate Cox regression, the risk model had a significant relationship with OS (HR = 1.7566, 95%CI (1.1988–2.5739), *P* < 0.005) (Fig. 4F). The multivariate analysis also indicated the risk model was an important factor correlated to OS (HR = 1.6734, 95%CI (1.1368–2. 4633), *P* < 0.01) (Fig. 4G). Furthermore, the heatmap of clinical features showed that the survival status was better in the low-risk group (Fig. 4H).

### Validation of the prognostic model in the Chinese Gliomas Genome Atlas (CGGA) databases

**Fig. 5:**
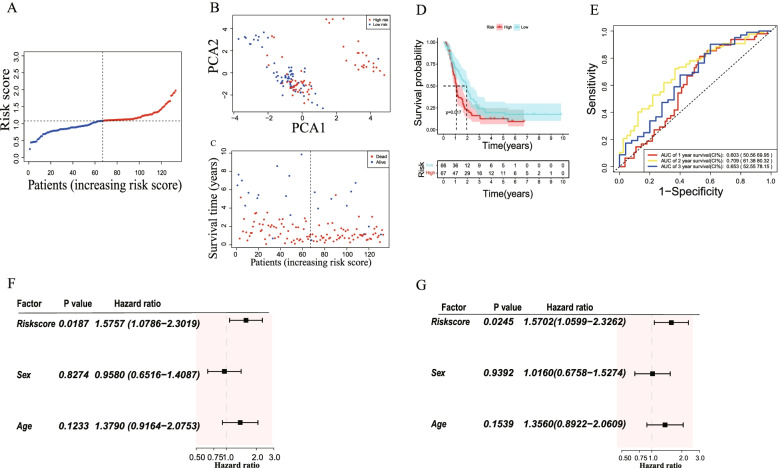
Validation of risk model in CGGA cohort. **A** Distribution of patients according to risk model in CGGA cohort. **B** Principal component analysis (PCA) plot of GBM patient based on risk model. **C** The survival status and time of patient with GBM. (Low-risk: on the left side of dotted line. High-risk on the right side of dotted line). **D** Kaplan-meier (KM) survival analysis of patients in low-risk and high-risk groups. **E** ROC curve for predicting efficiency of the risk model. **F** Univariate Cox regression analysis of risk score in CGGA cohort. **G** Multivariate Cox regression analysis of risk score in CGGA cohort

After screening out, 134 GBM patient’s samples with clinical informations were extracted from CGGA mRNAseq_693. According to the median risk score built in TCGA cohort, patients were classified into high-risk (66) and low-risk (67) groups (Fig. 5A). PCA analysis indicated that patients were separated well into two groups (Fig. 5B). The dot line plot showed that patients with low-risk exhibited better survival time and survival rate (Fig. 5C). Furthermore, Kaplan–Meier analysis also showed significant difference in survival status between two groups (*P* = 0.0175) (Fig. 5D). Time-dependent ROC curves indicated that the 1-year, 2-year, 3-year AUC were 0.603, 0.709, 0.653 respectively, suggesting satisfactory predicting efficacy of the risk score model (Fig. 5E).

To evaluate whether risk score was an independent risk factor, we utilized univariate and multivariable Cox regression analyses in CCGA cohort. In univariate Cox regression, the risk model had a significant relationship with OS (HR = 1.5757, 95%CI (1.0786–2.3019), *P* = 0.0187) (Fig. 5F). The multivariate analysis also indicated the risk model was an important factor correlated to OS (HR = 1.5702, 95%CI (1.0599–2. 3262), *P* = 0.0245) (Fig. 5G).

### Establishment and validation of nomograph

**Fig. 6:**
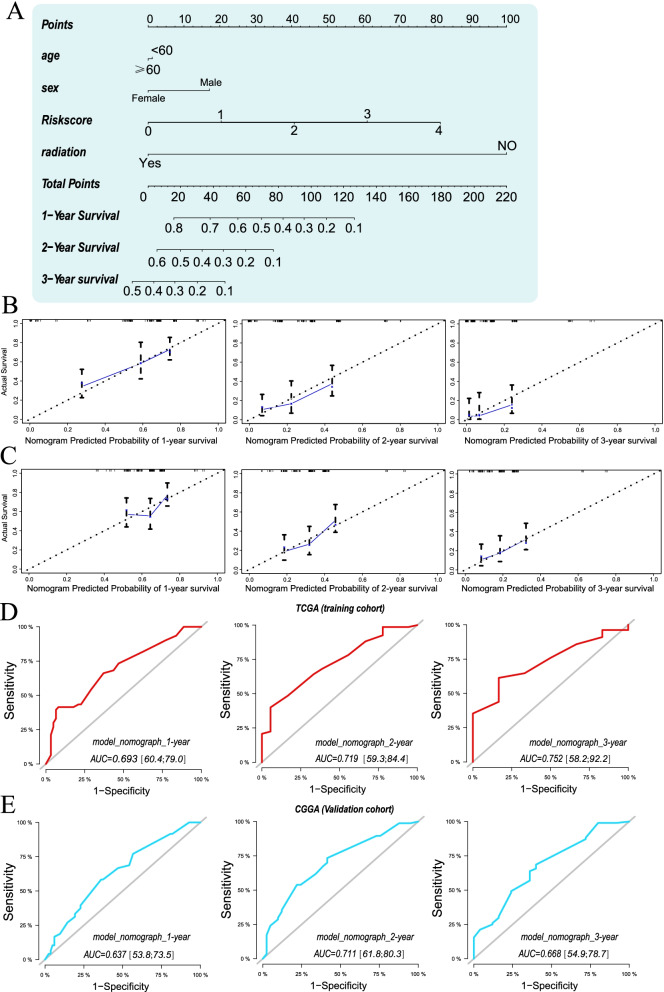
Establishment of nomograph for predicting 1-year, 2-year and 3-year survival probability. **A** Construction of prognostic nomograph to predict survival of GBM in TCGA cohort. **B** Calibration curves of the prognostic nomogram for predicting 1-year, 2-year and 3-year survival probability in the TCGA cohort. **C** Calibration curves of the prognostic nomogram for predicting 1-year, 2-year and 3-year survival probability in the CGGA cohort. The prognostic value of the nomogram evaluated by ROC curve in training (**D**) and validation cohort (**E**)

To construct a clinical-based method for predicting the prognosis of GBM patients, we established a nomograph based on 4 prognostic parameters including age, sex, radiation therapy, riskscore (Fig. 6A). The calibration plots indicated excellent agreement between the predicted and actual observation in both training and validation cohort (Fig. 6B, C). The AUC of nomograph in predicting 1-year, 2-year, 3-year survival were 0.693, 0.726, 0.731 respectively in TCGA cohort and were 0.637, 0.704, 0.681 respectively in CGGA cohort, indicating a favorable predictive ability of nomograph (Fig. 6D, E).

### The GO and KEGG enrichment analysis based on risk model

**Fig. 7:**
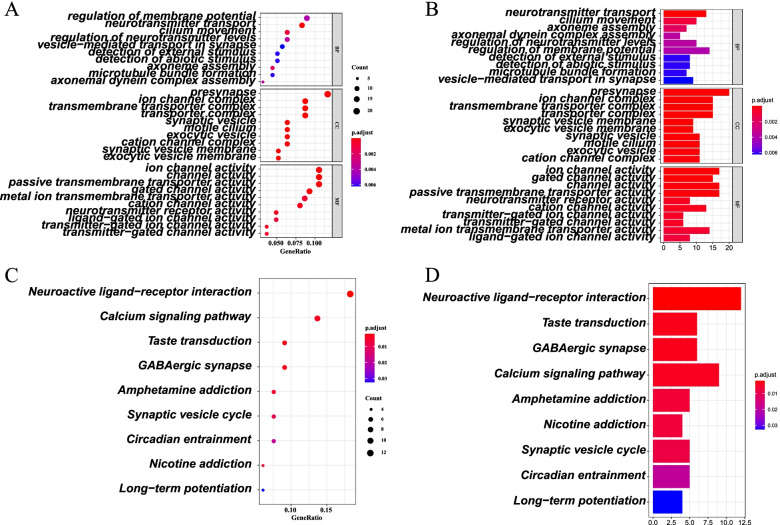
Functional enrichment of DEGs based on low-risk and high-risk groups. **A**, **B** Gene Ontology (GO) enrichment analysis of DEGs. **C**, **D** Kyoto Encyclopedia of Genes and Genomes (KEGG) pathway analysis of DEGs

To further clarify the pathway and the biological function of DEGs according to risk model, we performed Gene ontology (GO) enrichment analysis and Kyoto Encyclopaedia of Genes and Genomes (KEGG) pathway analysis on DEGs between low-risk and high-risk groups in TCGA cohort. First, we applied limma package in R language to identify 187 DEGs between two groups with the criteria FDR < 0.05 and |log2FC | ≥ 1. In 187 DEGs, 27 genes were downregulated in high-risk group, while the other 160 genes were upregulated. GO and KEGG enrichment analysis were applied on DEGs. According to GO analysis, DEGs based on risk model were mainly enriched in neurotransmitter transport, presynapse, ion channel activity (*P* < 0.005) (Fig. 7A, B). KEGG analysis showed that DEGs were closely associated with neuroactive ligand−receptor interaction and calcium signaling pathway (*P* < 0.01) (Fig. 7C, D).

### Evaluation of the immune activity between subgroups

**Fig. 8:**
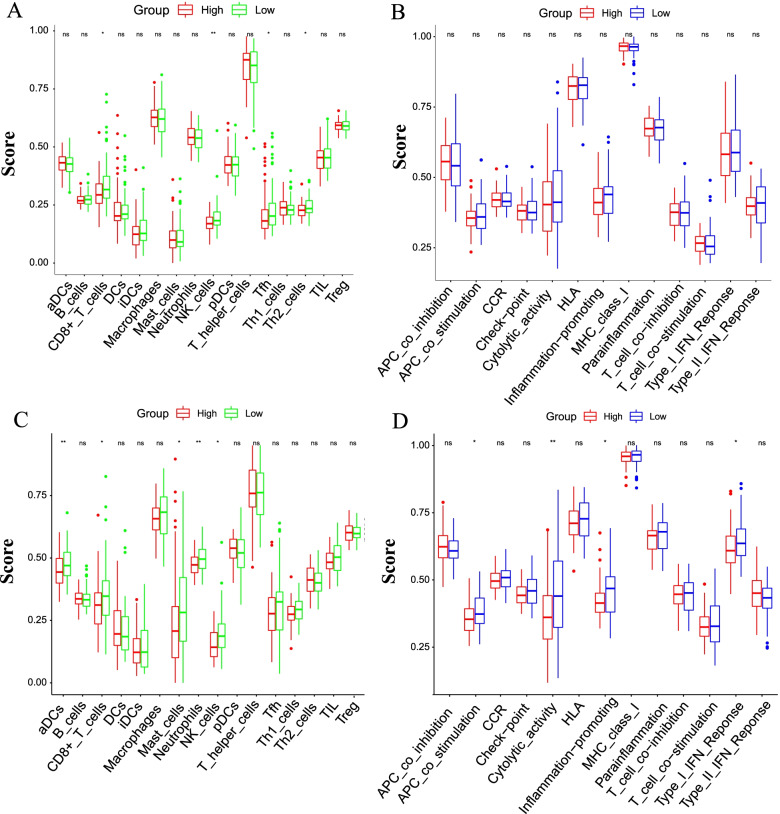
Differences of immune cells infiltration and immune pathway based on risk score. **A**, **B** ssGSEA scores of immune cells (A.16 immune cells) and immune pathway (B.13 immune pathways) in TCGA cohort. **C**, **D** single-sample gene set enrichment analysis **(**ssGSEA) scores of immune cells (A.16 immune cells) and immune pathway (B.13 immune pathways) in CGGA cohort. ^*^ *P* < 0.05, ^**^ *P* < 0.01, ^***^ *P* < 0.001

As immune micro-environmental abnormality plays an important part in oncogenesis, invasion and metastasis, we further evaluated the enrichment of 16 types of immune cells and 13 immune-related pathways in TCGA and CGGA in different risk score via single-sample gene set enrichment analysis (**ssGSEA**) package. As shown in TCGA boxplot, patients in high-risk group showed lower level of immune cells (CD8^+^ T cells, natural killer (NK) cells, follicular helper T cell (Tfh) cells, Th2 cell) than those in low-risk group (*P* < 0.05) (Fig. 8A), while the difference in immune pathway is not prominent (Fig. 8B).

**Fig. 9:**
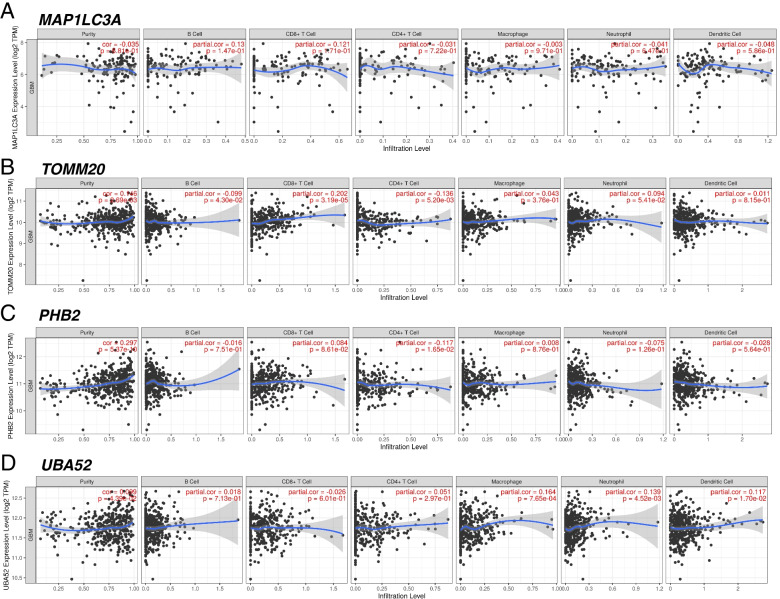
TIMER analysis of correlations between prognostic genes and immune cell infiltration. **A**, **B**, **C**, **D** Relationship between *MAP1LC3A, TOMM20, PHB2, UBA52* and immune cell infiltration

In CGGA cohort, high-risk group had lower infiltration of immune cells (CD8^+^ T cells, NK cells, activated dendritic cells (aDCs), Mast cells, Neutrophils), which was almost the same as TCGA (Fig. 8C). Additionally, APC co stimulation, cytolytic, inflammation promoting, Type I IFN response were enriched in low-risk group, indicated the low immune activity in high-risk group (Fig. 8D). To further analyze the relationship between mitophagy-related DEGs (*MAP1LC3A*, *TOMM20*, *PHB2*, *UBA52*) and immune cells, the TIMER 2.0 was applied (Fig. 9). As shown in Fig. 9, *TOMM20* was positively associated with CD8^+^ T cells (*P* < 0.0001) and negatively related to B cells and CD4^+^ cells (*P* = 0.043, 0.0052). *UBA52* had positively relationship with macrophage, neutrophil, dendritic cell (DC) (*P* = 0.00076, 0.0045, 0.017). *PHB2* had negatively relationship with CD4^+^ cells (*P* = 0.016). These findings suggested that the risk model genes were closely associated with immune infiltration.

### Evaluation of the tumor microenvironment between subgroups

**Fig. 10:**
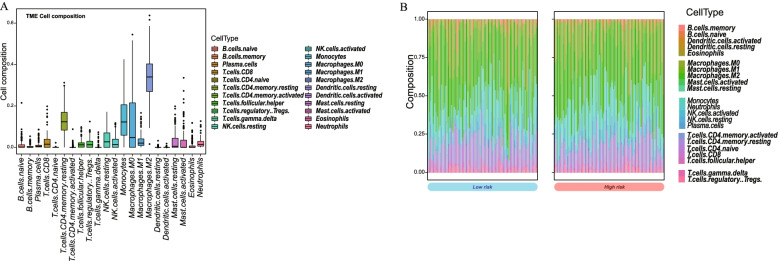
CIBERSORT analysis of immune cell composition in TCGA. **A** Overview of immune cell composition in GBM sample. **B** immune cell composition in high-risk and low-risk groups

Due to the close relationship between mitophagy-related risk model and immune activity, CIBERSORT was utilized to analysis the tumor microenvironment (TME) between low-risk and high-risk groups. The overview of 22 immune cell compositions in GBM samples was shown in Fig. 10A. The high-risk group had higher proportion of resting NK cells as well as plasm cells, while low-risk group had increased proportion of activated NK cells, indicating that the activation of NK cells regulated anti-tumor effects in low-risk group (Fig. 10B, C).

## Discussion

The heterogeneity of GBM and the lack of effective stage make it important to develop stable prognostic model. Our current study firstly analyzed the relationship between 51 mitophagy-related genes and the prognosis of GBM in TCGA cohort and constructed a mitophagy-related prognostic predicting model containing 4 genes (*MAP1LC3A*, *TOMM20*, *PHB2*, *UBA52*), which was validated well in external databases. Also, we then constructed a mitophagy-based nomograph with potent predicting value. Furthermore, GO and KEGG pathway enrichment analysis uncovered that the mitophagy-related genes are associated with synaptic activity, ion channel, neuroactive ligand-receptor interaction and calcium signaling. In addition, the mitophagy genes were closely related to immune infiltration and TME.

Mitophagy, an evolutionarily conserved programmed cell death essential for cellular homeostasis, is an autophagic response targeting damaged, dysfunction mitochondria. Despite numerous studies have pointed out that the autophagy plays a vital part in tumorigenesis, metastasis and drug resistance, limited studies reported about the role of mitophagy in cancer especially gliomas [6, 21]. In our research, we systematically combined mitophagy-related genes with survival data and constructed a 4-gene prognostic predicting model to predict OS in GBM patients, suggesting a strong relationship between GBM and mitophagy.

Four important mitophagy-related genes (*MAP1LC3A*, *TOMM20*, *PHB2*, *UBA52*) were identified in our study. Microtubule Associated Protein 1 Light Chain 3 Alpha (*MAP1LC3A*) encodes 2 different isoforms: *MAP1A* and *MAP1B*. *MAP1A* and *MAP1B* are two microtubule-related proteins mediating the physical interactions between microtubules and components of the cytoskeleton. Compelling studies reported that the expression of *MAP1LC3A* was suppressed in many tumor cells including GBM, indicating that it might be involve in the tumorigenesis of various cancers [22, 23]. Wang reported that upregulation of *MAP1LC3A* in GBM could predict poor prognosis [24]. Consistent with the studies above, we observed that the *MAP1LC3A* was downregulated in GBM samples compared to normal samples and it was enriched in high-risk group. Further Cox regression analysis showed that high expression of *MAP1LC3A* was related to poor survival, suggesting that dysregulation of mitophagy promoted the tumor progression. Translocase Of Outer Mitochondrial Membrane 20 (*TOMM20*) is a pre-protein receptor on the translocation complex of the mitochondrial outer membrane. According to previous studies, upregulation of *TOMM20* could be observed in many cancers such as hepatocarcinoma [25–28]. However, the expression of *TOMM20* was lower in GBM compared to normal samples in our study and it was highly expressed in low-risk group. One possible explanation is that *TOMM20* upregulated the CD8^+^ T cell, providing tumor-suppressive effect in our study based on TIMER analysis (Fig. 9). Prohibitin 2 (*PHB2*) is highly conserved protein mainly in mitochondria, nucleus and plasma membrane, which is required for Parkin-induced mitophagy in mammalian cells and cancer cell proliferation and adhesion [29]. Overexpression of *PHB2* had been reported in hepatocarcinoma, breast cancer as well as lung cancer [30–32]. *PHB2* was upregulated in GBM patients and enriched mainly in low-risk group in our study. The current discrepancy still urged further exploration on the two-edge sword effect of mitophagy. Ubiquitin A-52 Residue Ribosomal Protein Fusion Product 1 (*UBA52*) is a ubiquitin coding gene encoding a ubiquitin fusion protein which is comprised of ribosomal protein L40 at C-terminus and ubiquitin at the N-terminus [33]. Ubiquitin is closely related to cell cycle regulation and lysosomal degradation. Also, *UBA52* was observed in tumor tissues [34, 35]. It is reported that over-expression of *UBA52* induced cellular apoptosis in tumor tissue [36] and UBA52 was mainly enriched in low-risk group in our study.

Until now, the role of mitophagy in tumor has not been fully understood. On the one hand, inhibition of mitophagy suppressed the growth of glioblastoma cells [7]. On the other hands, lethal mitophagy can inhibited the proliferation of glioma [9]. According to data from TCGA cohort, about 56% mitophagy-related DEGs were downregulated with 44% upregulated in tumor sample, indicating the two-edges sword effect of mitophagy, which is consistent with the findings above. Furthermore, the *TOMM20* can facilitate ROS-induced pyroptosis and *PHB2* is associated with apoptosis, indicating the interaction and coexistence of different programmed cell death as tumor grows [37].

Through LASSO and Cox regression analysis, we then constructed a mitophagy-related risk model and nomograph to predict prognosis of GBM. Based on risk model, patients were divided into high-risk and low-risk group. As patients with high-risk developed poor prognosis, more aggressive methods and closer follow-up time interval are required, indicating the risk model offer precise individualized treatment in clinical practice. Nomograph is characterized by intuitive visual presentation in guiding clinical practice and we established a mitophagy-related nomograph for the first time with excellent predictive performance, which is superior than conventional WHO stage.

To further analyze the functional enrichment between low-risk and high-risk groups, GO and KEGG pathway analysis were conducted. Go analysis suggested that the DEGs mainly involved in synaptic activity and ion channel, which is consistent with previous findings that synaptic activity drives the progression of gliomas and ion channel is closely related to the proliferation, metastasis, invasion of GBM [38, 39]. KEGG pathway analysis indicated that the DEGs between 2 subgroups mainly enriched in neuroactive ligand-receptor interaction as well as calcium signaling. Previous studies demonstrated that neuroactive ligand-receptor interaction plays an important part in development of GBM [40, 41]. A comprehensive analysis conducted by Pal J found that GBM patients with defective neuroactive ligand-receptor interaction had poor prognosis [40]. Calcium signaling was closely related to the tumorigenesis, progression of GBM [42]. These findings suggested that the 4-gene risk model might regulate tumor progression through the pathways or processes above.

As previous studies suggested that immune infiltration played a crucial role in the prognosis of patients with glioma, we further analyzed and demonstrated that the immune filtration and immune pathway were statistically different between 2 subgroups [43]. It is reported by recent studies that GBM patient with numerous CD8^+^ T cells tend to have better survival, while activated NK cells predict better prognosis in GBM [44]. In our study, the level of CD8^+^ T cells, NK^+^ cells, Tfh cells and Th2 T cells were all downregulated in high-risk group, suggesting a suppressive immune infiltration in patients with poor prognosis. In addition, the prognostic gene *TOMM20* is positively corelated to CD8^+^ T cells in TIMER analysis (Fig. 9), suggesting a close link between mitophagy and immune infiltration. The enigmatic and sophisticated association linking immunity with mitophagy are being gradually uncovered with the progress of the experimental research. One study from Paul K revealed that mitophagy could trigger the proliferation of CD8^+^ cell to improve prognosis in cancer [45]. Another study from Alejandro López-Soto suggested that the function of NK cell can be modulated by mitophagy [46]. Furthermore, the immune pathway analysis mainly focused on APC costimulation, inflammation-promoting, type I IFN response pathway. Finally, CIBERSORT analysis revealed that the primary immune cells enriched in the low-risk group were activated NK cells, suggesting the crucial role of activation of NK cells in mediating better prognosis. Taken all together, our studies indicated that the mitophagy-related genes modulated immune microenvironment and affected the prognosis.

There are some limitations in our study. First, the clinical data from TCGA are incomplete such as therapy and therapeutic effect, which might provide clues on biomarker of treatment. Second, validation of risk signature is best carried out *in vivo* or well-established study, as external databases often have bias on race or area.

## Conclusion

In summary, we utilized 4 mitophagy-related genes to construct a risk model that accurately predicts the prognosis of GBM patients. Our findings suggested the crucial role of mitophagy in GBM, which might be related to tumor immune microenvironment modulation. Further studies are needed to verify these results *in vitro* and *in vivo*.

## Supplementary Information

**Additional file 1.**

**Additional file 2.**

**Additional file 3.**

**Additional file 4.**

## Data Availability

The datasets used and/or analysed during the current study available from the corresponding author on reasonable request.

## Abbreviations

GBM: Glioblastoma multiforme
DEGs: Differentially expressed genes
LASSO: Least absolute shrinkage and selection operator
TME: Tumor microenvironment
PPI: Protein protein interaction
PCA: Principal component analysis
AUC: Area under the curve
KEGG: Kyoto Encyclopaedia of Genes and Genomes
ssGSEA: Aingle-sample gene set enrichment analysis
GO: Gene ontology
STRING: Search Tool for the Retrieval of Interacting Genes
NK: Natural killer
Tfh: Follicular helper T cell
MAP1LC3A: Microtubule Associated Protein 1 Light Chain 3 Alpha
TOMM20: Translocase Of Outer Mitochondrial Membrane 20
PHB2: Prohibitin 2
UBA52: Ubiquitin A-52 Residue Ribosomal Protein Fusion Product 1
