# Supplementary figures and images for: Development and validation of a novel mitophagy-related gene prognostic signature for glioblastoma multiforme
Source: BMC Cancer. 2022 Jun 13;22:644. doi: 10.1186/s12885-022-09707-w (PMC9190154; doi:10.1186/s12885-022-09707-w)

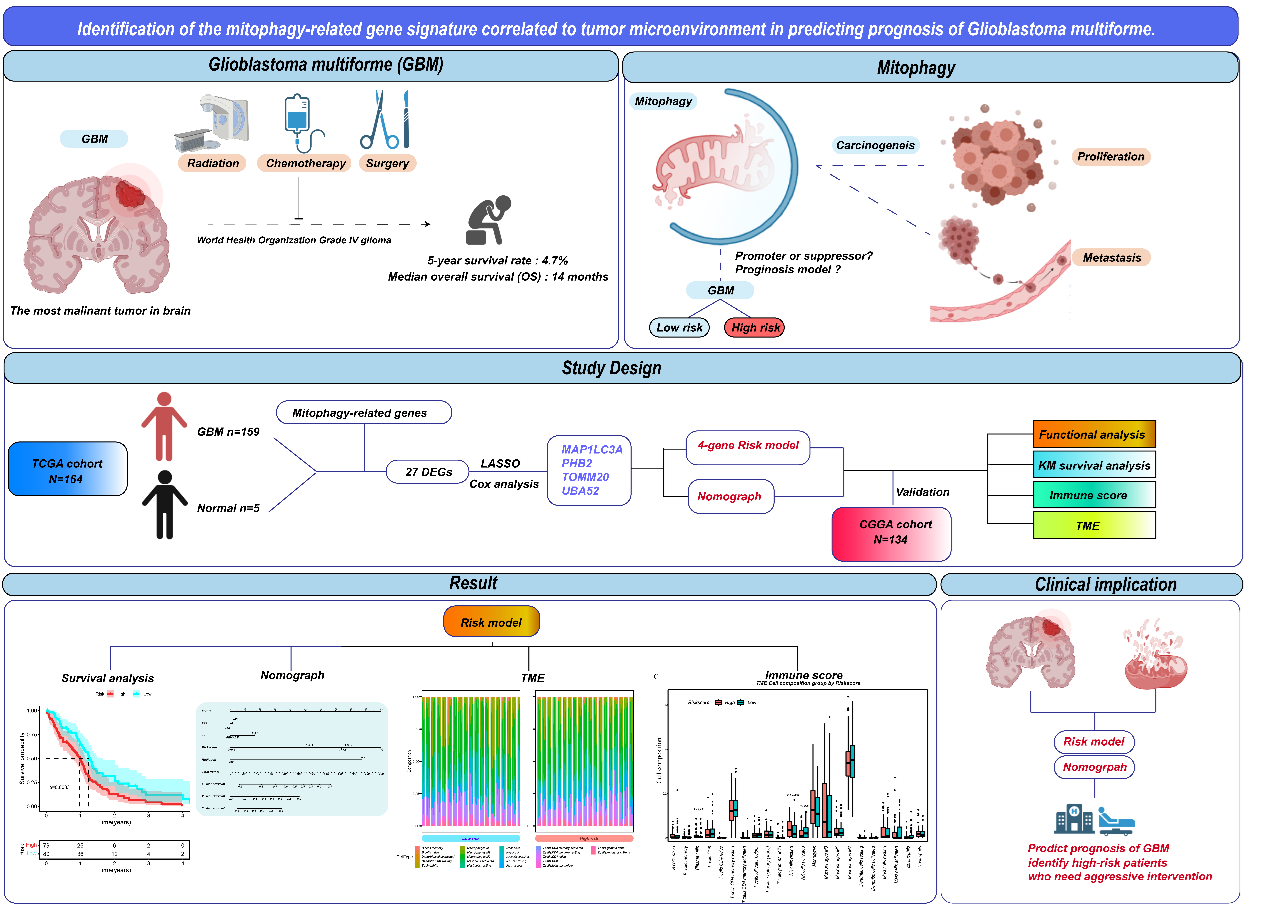

Supplement: Supplementary file 2 — Additional file 2. [file 12885_2022_9707_MOESM2_ESM.tif]

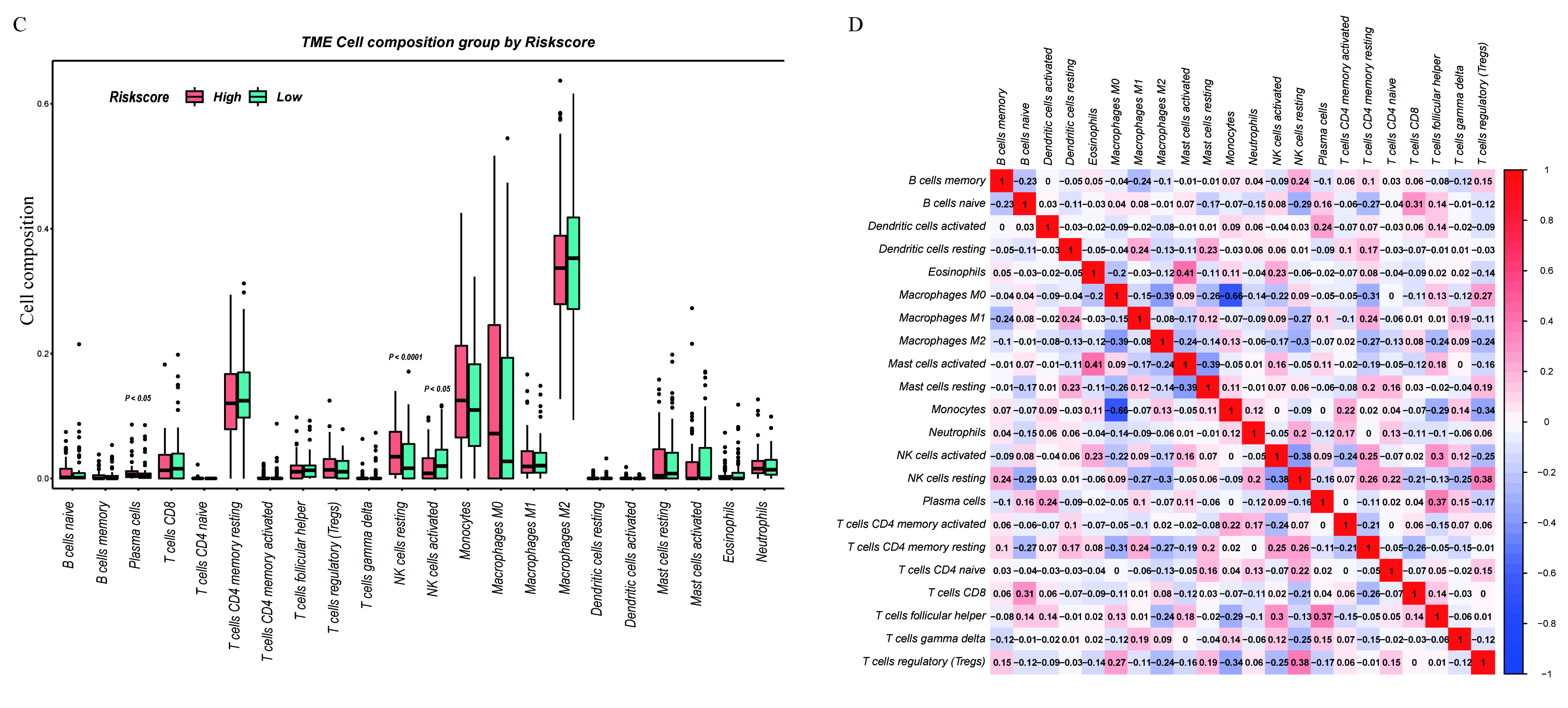

Supplement: Supplementary file 4 — Additional file 4. [file 12885_2022_9707_MOESM4_ESM.tif]
